# AGE OF MUSIC ACQUISITION IMPACTS MEMORY FUNCTIONING IN OLDER ADULT MUSICIANS

**DOI:** 10.1093/geroni/igz038.2417

**Published:** 2019-11-08

**Authors:** Jessica V Strong, William Milberg, Regina McGlinchey, Elizabeth Leritz

**Affiliations:** VA Boston Healthcare System, Boston, Massachusetts, United States

## Abstract

Music playing is an involved activity, activating many areas of the brain and relying on integration of multiple cognitive processes simultaneously. Older adult musicians have been found to experience some cognitive benefits compared to non-musicians, seemingly related to their musical training. However, we still do not understand what factors of musical training may be driving these differences. The current study sought to isolate age of acquisition from “dose” of playing (i.e., amount of time spent playing) to explore music learning as a skill acquired during a sensitive period. Participants (n=48) were middle aged and older adults who self-reported on musical experiences, demographics, and underwent extensive neuropsychological assessment of all major domains. The sample was divided into Early Age of Acquisition (≤9 years old), Late Age of Acquisition (>9), and Non-Musicians. Results showed that musicians who began formal training at the age of 9 or younger, had significantly higher scores on tests of verbal memory (California Verbal Learning Test – II: Immediate Recall – p = 0.04, partial η2 = 0.14, Short-Delay Free Recall - p = 0.03, partial η2 = 0.16, Long-Delay Free Recall - p = 0.03, partial η2 = 0.15). Results are discussed in the context of a sensitive period for acquiring musical education, and implications of these results on cognitive aging.

